# Nevoid Basal Cell Carcinoma Syndrome: *PTCH1* Mutation Profile and Expression of Genes Involved in the Hedgehog Pathway in Argentinian Patients

**DOI:** 10.3390/cells8020144

**Published:** 2019-02-11

**Authors:** Maria Florencia Martinez, Maria Vanesa Romano, Alfredo Pedro Martinez, Abel González, Carolina Muchnik, Fernando Miguel Stengel, Luis Daniel Mazzuoccolo, Pablo Javier Azurmendi

**Affiliations:** 1 Laboratorio de Nefrología Experimental y Bioquímica Molecular, Instituto de Investigaciones Médicas “Alfredo Lanari”, Facultad de Medicina, Universidad de Buenos Aires, 1427 Buenos Aires, Argentina; martinez.mflorencia@gmail.com (M.F.M.); caromuch@hotmail.com (C.M.); 2 Departamento de Análisis Clínicos, Centro de Educación Médica e Investigaciones Clínicas “Norberto Quirno”, 1431 Buenos Aires, Argentina; vromano@cemic.edu.ar (M.V.R.); amartinez@cemic.edu.ar (A.P.M.); 3 Unidad de Cirugía de MOHS, Departamento de Cirugía de Cabeza y Cuello, Instituto de Oncología “Ángel H. Roffo”, Facultad de Medicina, Universidad de Buenos Aires, 1408 Buenos Aires, Argentina; abelgo@gmail.com; 4 Buenos Aires Skin, 1055 Buenos Aires, Argentina; fernando@stengeldermato.com.ar; 5 Servicio de Dermatología, Hospital Interzonal General de Agudos “Eva Perón”, 1650 Gral. San Martin, Argentina; luis.mazzuoccolo@hospitalitaliano.org.ar; 6 Servicio de Dermatología, Hospital Italiano de Buenos Aires, 1199 Buenos Aires, Argentina

**Keywords:** Gorlin–Goltz syndrome, *PTCH1* mutation, Hedgehog pathway, basal cell carcinoma

## Abstract

Nevoid basal cell carcinoma syndrome (NBCCS) is an autosomal dominant disorder characterized by multiple basal cell carcinomas (BCC), mainly caused by *PTCH1* gene mutations. Our current study aimed to establish (1) *PTCH1* germinal and somatic mutational status, (2) component and Hedgehog (HH) pathway targets gene expression patterns, and (3) profile variations according to the genetic background in BCC and normal surrounding skin (NSS). We collected 23 blood and 20 BCC patient samples and analyzed the *PTCH1* gene using bidirectional sequencing and multiplex ligation-dependent probe amplification. Quantitative PCR was used to determine the mRNA expression levels of *PTCH1*, *SMO*, *GLI3*, and *CCND1* in paired samples of BCC and NSS from 20 patients and four non-NBCCS skin controls (C). Our analyses identified 12 germline and five somatic sequence variants in *PTCH1*. mRNA levels of *PTCH1*, *SMO,* and *GLI3* were higher in NSS compared to C samples, reaching maximum values in BCC samples (*p* < 0.05). NSS with *PTCH1* germline mutations had modified *SMO,* *PTCH1*, and *GLI3* mRNA levels compared to samples without mutation (*p* < 0.01). Two *PTCH1* mutations in BCC led to an increase in *PTCH1*, *SMO,* and *GLI3*, and a decrease in *CCND1* mRNA levels (*p <* 0.01 vs. BCC with germline mutation only). These results indicate that besides *PTCH1*, other genes are responsible for NBCCS and BCC development in a population exposed to high UV radiation. Additionally, the mutational events caused increased expression of HH-related genes, even in phenotypically normal skin.

## 1. Introduction

Nevoid basal cell carcinoma syndrome (NBCCS; OMIM #109400)—also known as the Gorlin–Goltz Syndrome—is a multisystem genetic disorder with an autosomal dominant inheritance pattern, complete penetrance, and variable expression [1]. NBCCS patients are characterized by numerous malformations and a high susceptibility for developing multiple basal cell carcinomas (BCC)—the most common type of cancer worldwide—at an early age. *PTCH1* is the human homolog of the *Drosophila segment polarity* gene, with 23 exons that encode a transmembrane glycoprotein composed of 1447 amino acids, and is responsible for most NBCCS cases. PTCH1 acts as a receptor in the Hedgehog signaling pathway [2,3]. Its ligands, Hedgehog (HH), are morphogens that physiologically drive the inhibitory signaling effects of PTCH1 over smoothened, which in turn activates the GLI effectors. GLI proteins are transcription factors that stimulate their target genes to induce cell cycle effects. These proteins have alternate activating and repressing functions depending on HH concentration and gradient [4]. In several cell types, the HH pathway activation increases the expression of key regulators from the G1/S and G2/M cell cycle phases, promoting the transition from the quiescent to the proliferative state. Mutations in *PTCH1* cause constitutive activation of the HH pathway, which in turn leads to the development of NBCCS [5].

Ponti et al. proposed that normal cells become tumorigenic via the Knudson’s two-hit mechanism, based on evidence showing loss of heterozygosity in genetic studies in human odontogenic keratocysts [6]. Based on this hypothesis, the addition of somatic mutations to inherited germline mutations was associated with the development of these and other tumors, such as BCC and meningiomas [1]. It has also been described that the progressive allele inactivation of *PTCH1* over-activates the HH pathway, leading to cell cycle progression [7]. However, systematic analysis of pathway over-activation and the presence of mutations in these patients have not been entirely clarified. Therefore, we systematically looked for mutations in the *PTCH1* gene in peripheral blood and BCC tissue of patients with NBCCS. In addition, we quantified the expression of Hedgehog pathway genes and its effectors in BCC and phenotypically normal adjacent tissue, to evaluate expression profile variations according to the mutational status.

## 2. Materials and Methods

Twenty-three patients from 20 unrelated families, who complied with the diagnostic criteria defined by Kimonis for NBCCS, were included in the study [8].

Genomic DNA and total RNA were isolated from BCC and normal surrounding epithelial tissue (NSS) samples following a routine biopsy. Genomic DNA was also extracted from blood samples obtained by venipuncture, using 0.5% EDTA as an anticoagulant.

### 2.1. Extraction of Nucleic Acids

Genomic DNA and total RNA were sequentially isolated using an SV Total RNA Isolation System kit (Promega Corporation, Madison, WI, USA) [9], whereas DNA from blood was obtained using a High Pure PCR Template Preparation kit (Roche Applied Bioscience, Roche Diagnostic GmbH, Mannheim, Germany), following the manufacturers’ instructions. The DNA and RNA samples were stored at −20 °C and −70 °C, respectively.

### 2.2. Mutation Search

#### 2.2.1. PTCH1 Gene Sequencing

Blood (23/23) and BCC (20/23) samples were used as starting materials to amplify the 23 exons from the *PTCH1* coding sequence. The experiment was conducted in 20 PCR reactions using published primer sequences [10,11], following the protocols described in References [12,13]. The PCR conditions were as follows: Initial denaturalization at 95 °C for 5 min; followed by 35 cycles of denaturalization at 95 °C for 30 s; annealing at a specific temperature depending on the primers for 30 s; elongation at 72 °C for 55 s for fragments under 700 bp, or for 90 s for longer fragments; and final elongation at 72 °C for 5 min. Blood DNA samples from four healthy subjects were included as controls in each experiment. 

Bidirectional sequencing was conducted in an automated sequencer ABI PRISM^®^ 310 Genetic Analyzer (Applied Biosystems, Foster City, CA, USA), using BigDye™ Terminator v3.1 Cycle Sequencing kit (Applied Biosystems), according to our previously published procedure [12,13]. We used 5–10 ng of the material purified using PureLink Quick Gel Extraction kit (Invitrogen, Life Technologies, Carlsbad, CA, USA).

#### 2.2.2. PTCH1 Gene Analysis using Multiplex Ligation-dependent Probe Amplification (MLPA)

The detection of major sequence duplications or deletions was performed via MLPA using the commercial kit P067-B1 PTCH1 (MRC—Holland, The Netherlands), following published procedures [12,13]. Each MLPA reaction generated a combination of 23 amplified fragments in one single PCR, which were identified and quantified using capillary electrophoresis with an ABI PRISM^®^ 310 Genetic Analyzer (Applied Biosystems). Patterns of the generated peaks were assessed using the Coffalyser.Net software [14]. The heterozygous deletion profile showed a reduction in 30–50% of the peak area compared to controls, whereas profile duplication increased the peak area by 35–55%.

#### 2.2.3. Genetic Variant Analysis

The functionality of variants that were not synonymous was predicted using SIFT (http://siftdna.org/www/Extended_SIFT_chr_coords_submit.html) [15], and PolyPhen2 (http://genetics.bwh.harvard.edu/pph2/) [16] software programs. In the case of variants that changed their nucleotide sequence, Mutalyzer version 2.0.29 (https://mutalyzer.nl/) was used to assess this change and verify the nomenclature [17]. Variants, including a change in nearby regions or in splicing regions, were assessed using the Human Splicing Finder Analyze Mutation software (http://www.umd.be/HSF/) [18].

Variants found in both patients and controls, which were predicted as nonpathogenic using bioinformatics analysis, were considered benign. Whereas, variants that did not exist in control samples, and were predicted as pathogenic were considered mutations. Using these analyses results, a mutation was assigned to a germline status if it was found in the same patient’s BCC and blood samples. On the other hand, a somatic nature was assigned to the mutations found only in patient tumors.

### 2.3. Gene Expression Analysis

*PTCH1* (gene ID: 5727), *SMO* (ID: 6608), *GLI3* (ID: 2737), and *cyclin D1* (*CCND1*, ID: 595) gene expressions were evaluated by quantitative real-time PCR, using total RNA from BCC and NSS of NBCCS patients, and normal skin (NS) from four healthy subjects, as starting material. One NSS and one BCC sample per subject were obtained from 20 out of 23 patients. RNA was retrotranscribed to cDNA using Illustra Ready-To-Go RT-PCR Beads kit (GE Healthcare UK Limited, Little Chalfont, UK), as described previously [9,19]. Real-time PCR was performed using LightCycler^®^ FastStart DNA Master SYBR Green I kit (Roche Applied Bioscience, Roche Diagnostic GmbH, Germany) with a LightCycler^®^ 2.0 carrousel system (Roche Diagnostic GmbH, Germany). The primers were designed with the Primer 3 software (http://bioinfo.ut.ee/primer3-0.4.0) [20], using NM_000264.3, NM_005631.4, NM_000168.5, and NM_053056.2 as the reference target mRNA sequences for *PTCH1*, *SMO*, *GLI3*, and *CCND1*, respectively; the *GAPDH* gene was employed as a housekeeping normalizer gene using the primers reported by Rubio et al. [21]. The primer sequences are available upon request. The real-time PCRs were run in duplicate for each sample, and the gene expression was calculated according to Pfaffl [22], where Ct represents the first cycle at which the output signal exceeds the threshold level.

### 2.4. Statistical Analysis

Continuous variables are shown as means ± standard error or percentage/frequency, as applicable. A *p*-value < 0.05 was considered significant. The Chi-square test was used for dichotomous comparisons, and the Mann–Whitney test or Student t-test was used for continuous comparisons. The results were analyzed using a factorial MANOVA, with the main variables (mRNA expression of *PTCH1*, *SMO*, *GLI3,* and *CCND1*) representing the dependent outcomes to be analyzed. After finding the significant MANOVA for the main variables, or for their respective interactions, differences between the groups were explored according to the dependent variable. Statistical analyses were conducted using IBM SPSS Statistics 21.0.0.0.

Ethical approval for this study was obtained from each participant hospital-authorized Ethics Committees. All patients provided written informed consent. The study was conducted according to the Declaration of Helsinki Principles.

## 3. Results

Due to the autosomal dominant nature of NBCCS, our mutation search focused on the 20 index cases from each family. The most frequent clinical manifestations (Table 1) were BCCs (18/20), odontogenic cysts (18/20), and palmoplantar pits (18/20). Among the infrequent findings, we found two patients without BCC—one of them with agenesis of the corpus callosum, and the other with NBCCS with concomitant autosomal dominant polycystic kidney disease [12,13].

### 3.1. PTCH1 Gene Mutations

*PTCH1* gene sequencing and MLPA analysis identified 12 germinal and five somatic mutations (Table 2), distributed throughout the gene. Among these mutations, 10 were frameshift mutations causing premature stop codons; three were missense changes resulting in harmful or pathogenic effects, as shown in the analysis performed by the prediction software; c.1068-2A>G led to a cryptic splice site abolition, according to the Human Splicing Finder Analyze Mutation prediction, which resulted in a frameshift with a premature stop codon at 16 bp from the mutation site; and one mutation generated an amino acid change in a premature stop codon.

#### 3.1.1. Localization of Mutations Found in NBCCS Patients

According to the protein structure, based on data from the ProtParam database (https://web.expasy.org/protparam/), five of the 12 germline mutations (c.513del, c.573C>G, c.652dup, c.1068-2A>G, and c.1286del) were located at the first extracellular loop, two at the first cytoplasmic loop and the sterol-sensing domain (c.1375_1399dup and c.1392_1405del), and two at the fourth extracellular loop (c.2309_2312del and c.2677del). The remaining mutations were located at the amino-terminal end (c.290del), and the third and fifth intracytoplasmic loops (c.2012dup and c.3277A>G, respectively).

**Figure 1:**
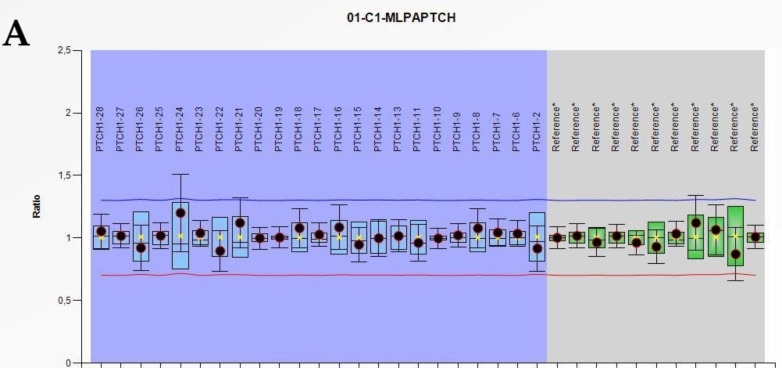

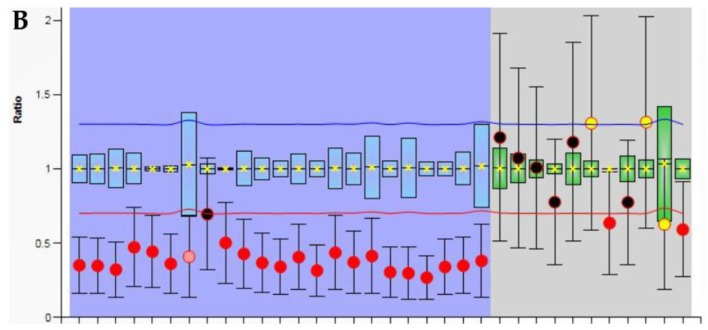
Representations of wild type (**A**) and allelic *PTCH1* deletion (**B**) in the BCC of patient SG12 obtained from multiplex ligation-dependent probe amplification (MLPA) analysis. The blue and green boxes represent the interquartile range of normal (N = 3) values for 23 *PTCH1* exons and 11 reference non-*PTCH1* probes, respectively, of the P067-B1 *PTCH1* kit. The blue and red lines represent duplication and deletion cut-off values, respectively. Patient median values for each probe and their standard deviations are shown as circles filled with **red** (abnormal), **yellow** (indeterminate), or **black** (normal).

With respect to somatic mutations, we identified a second somatic mutation in two patients that resulted in the loss of *PTCH1* gene heterozygosity. In patient SG12, the MLPA technique identified the loss of an entire allele (Figure 1), and two somatic mutations (c.3580C>T and c.3587C>T) were located at the protein carboxy-terminal end. No germline-independent somatic mutations were detected in the *PTCH1* gene. According to our results (Table 2), five patients showed germline and somatic mutations in the same gene, which supports the Knudson’s two-hit hypothesis for tumor development [27].

In conclusion, 83% of the detected germline mutations generated a truncated protein. Among the germline mutations detected in our study population, three have been previously described [23,24,25,26], and nine represent novel mutations, which have not been identified in other families or the NBCCS-associated bibliography.

#### 3.1.2. Analysis of NBCCS Families with PTCH1 Mutations

**Figure 2:**
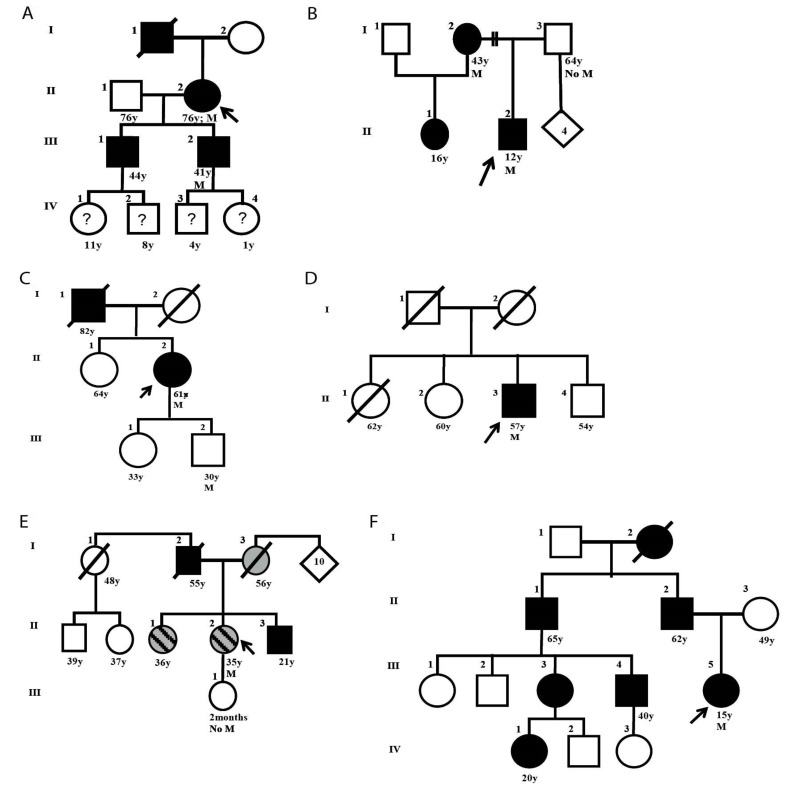
Genealogy tree of families with nevoid basal cell carcinoma syndrome (NBCCS) and known germline mutation: (**A**) SG6 family, (**B**) SG7 family, (**C**) SG12 family, (**D**) SG13 family, (**E**) SG20 family, and (**F**) SG21 family. The black arrows indicate the studied proband. Symbols filled in black indicate affected members with clinical manifestations. Symbols with “?” indicate that no clinical diagnosis was made. Symbols filled in grey indicates the family member affected by Autosomal Dominant Polycystic Kidney Disease (ADPKD) whereas the stripped ones indicate the family members affected by NBCCS and ADPKD. M: Mutation found in *PTCH1*; No M: No mutation in *PTCH1*.

The 12 patients with known germline mutations were re-called for update interviews to receive feedback on their analyses, to obtain more family data, and to offer the possibility of extending the genetic study to their relatives. Family trees in Figure 2 correspond to a detailed assessment of the patients enrolled and their relatives. Using this analysis, we identified two patients with singular clinical attributes that were directly or indirectly linked to the primary cilium [12,13].

Patient SG7 and his family were examined, as this patient’s neurologic image did not show the corpus callosum (a criterion that is not particularly sought in these patients), along with a negative family history of BCC (one of the typical criteria for NBCCS). The discussion involved the HH pathway in the primary cilium, which participates in the embryonic commissural plate’s development pattern that originates from the corpus callosum [12]. Patient SG20, who inherited NBCCS from her paternal side without BCCs at the time of enrollment, presented an inheritance pattern of autosomal dominant polycystic kidney disease via the maternal line. Polycystic kidney disease is a ciliopathy, since the anomalous protein responsible is also located in the primary cilium, as are the HH pathway components [13].

### 3.2. Hedgehog Pathway Gene Expression

To investigate the effects of detected mutations on the Hedgehog pathway gene expression, we quantified the expression of genes *PTCH1*, *SMO*, *GLI3,* and *CCND1* in BCC (N = 20), NSS samples (N = 20) from NBCCS patients, and NS from healthy controls (N = 4).

**Figure 3:**
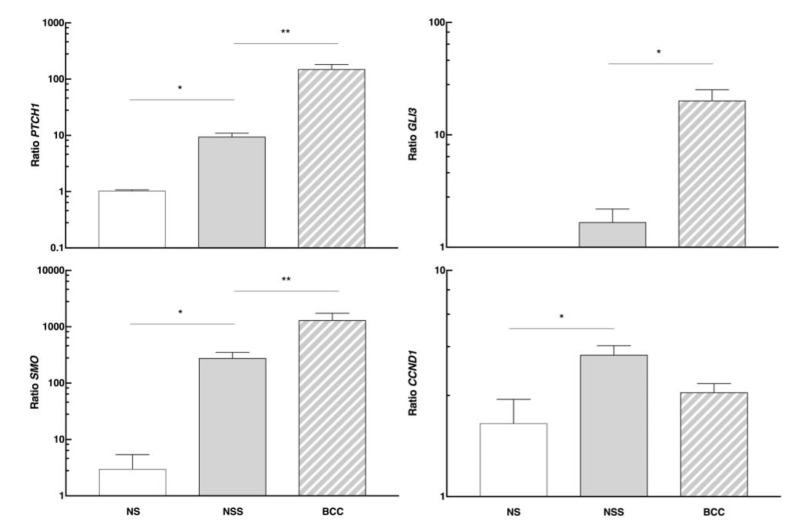
mRNA levels of *PTCH1*, *SMO*, *GLI3*, and *CCND1* genes studied in normal skin (NS) of controls, as well as in the normal surrounding skin (NSS) and basal cell carcinomas (BCC) of NBCCS patients. * *p* < 0.05 and ** *p* < 0.03.

*PTCH1*, *SMO*, and the *GLI3* effector—whose expression is almost undetectable in NS—had an increased expression in patients’ NSS compared to the NS group (Figure 3). This difference was even more pronounced in the BCCs, indicating overexpression of the pathway in this tissue. Furthermore, we analyzed *CCND1* expression levels as one of the transcriptional targets in the HH pathway, and observed an increase in NSS of patients compared to the NS control group.

#### PTCH1 Gene Mutations Affecting the Hedgehog Pathway Gene Expression

**Figure 4:**
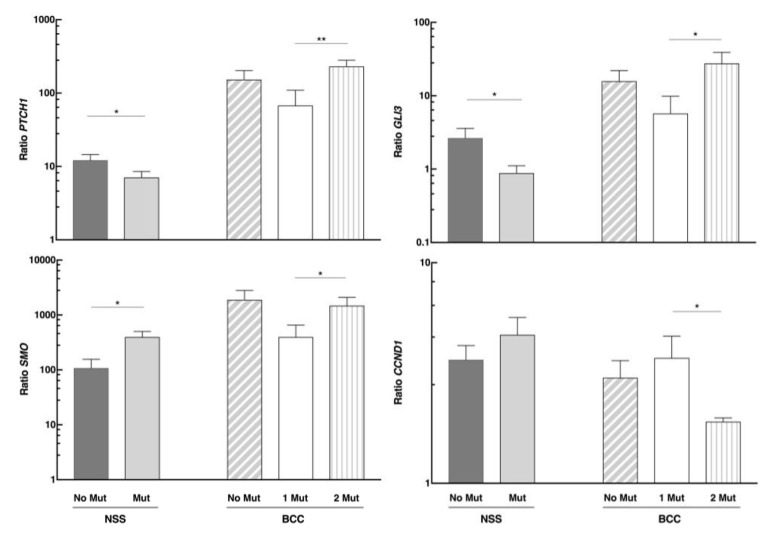
mRNA levels of *PTCH1*, *SMO*, *GLI3*, and *CCND1* genes in normal surrounding skin (NSS) and basal cell carcinomas (BCC) of NBCCS patients, according to their genetic status. No Mut: No mutation found in *PTCH1*; Mut: Tissue with the germline mutation in *PTCH1*; BCC 1 Mut and 2 Mut: Tissue with germline or germline plus somatic mutations in *PTCH1*, respectively. * *p* < 0.05 and ** *p* < 0.01.

We analyzed the same gene expression in different tissue according to our patients’ *PTCH1* genetic status. The expression of this pathway-repressing gene decreased in the NSS of the group with a germline mutation in *PTCH1* (N = 11) compared to those without the mutation (N = 9) (Figure 4). The same expression pattern was observed when we studied the *GLI3* effector. However, *SMO* had the opposite effect—its expression in the NSS of the group with *PTCH1* mutation was higher than in those without mutation. In the case of *CCND1*, there was no significant change in the NSS with respect to the genetic status.

Expression levels of *PTCH1*, *SMO,* and *GLI3* were higher in tumors with germline and somatic mutations in *PTCH1* compared to those with germline mutations only, whereas *CCND1* levels reflected the opposite behavior. Therefore, it is clear that the second mutational event further stimulates the expression of the pathway participants.

## 4. Discussion

This study provides a new mutational analysis of the *PTCH1* gene, distinguished by its germinal and somatic status, and the Hedgehog pathway gene expression profile in Argentine patients with NBCCS. We describe herein the largest Latin American cohort that belongs to a population exposed to larger UV radiation, due to its geographical location. We also found that the Hedgehog pathway was found to be active in these patients, both in their healthy skin and in their BCCs. Furthermore, the co-occurrence of two mutations modified these expression levels, suggesting that the cells genetic status affects the phenotypic destination of different tissues.

A significant proportion of patients did not have *PTCH1* mutations (even after an extensive mutation search), which supports the hypothesis of NBCCS’s multigenic nature. This hypothesis has already been proposed by other authors [11], and indicates the involvement of defects in genes other than *PTCH1*. The percentage of germline mutations detected for the *PTCH1* gene, within the range described in other series, also highlights the genetic heterogeneity of the syndrome [2,26]. Genetic diagnosis, despite its cost, is a valuable tool for affected families, particularly in those cases with inconclusive clinical signs. It, therefore, becomes essential for identifying other carriers in the family to aid clinicians when giving genetic advice. For example, genetic diagnosis may help the patient, and provide parents with a strong awareness of the severe consequences that ultraviolet radiation and/or X-ray exposure may have from the moment of the diagnosis.

Likewise, the somatic mutation discovery made in 5/20 patients was within the range reported by Pellegrini [28], whereas the lack of somatic mutations in half the BCCs of patients, who had a germline mutation, pointed towards other target genes as potentially being responsible for tumorigenesis. In this respect, germline mutations in other genes of the HH pathway have been detected, such as *SUFU* and *PTCH2*, in isolated reports of families with NBCCS [29,30]. Moreover, somatic mutations in *SMO* and *SUFU* have been found in sporadic BCCs [28].

The main objective of our study was to determine the underlying tumorigenic mechanisms in BCCs of patients with NBCCS. Unden et al. formulated the two-hit theory for tumor suppressor genes [31], which was later applied to odontogenic cysts [32,33,34]. In our patient groups, the proposed mechanism explains tumorigenesis in ~50% of the tumors with a known germline mutation. Thus, tumor suppressor genes-associated mutations phenotypically behave as recessive, meaning that to observe their effect it is necessary for both copies of the gene to be inactive in the cell [31,35]. However, in a particular group of tumors, we cannot dismiss the hypothesis of haploinsufficiency, as has been reported in some brain tumors [36,37]. It should be noted that we only detected somatic *PTCH1* mutations in patients who had a germline mutation in that gene, suggesting that allelic instability leads to somatic mutations in the wild allele, promoting normal tissue progression to tumor tissue. Likewise, we cannot rule out that those patients with germline *PTCH1* mutations could have somatic mutations in other genes of the HH pathway or another pathway involved in cell proliferation or differentiation.

Gene expression profile analysis showed that the skin of patients with NBCCS exhibited activation of the HH pathway even when phenotypically normal, and levels reached a maximum after malignant transformations. This finding conclusively showed fundamental involvement of HH in the syndrome, and changes at the level of expression. *PTCH1* and *GLI3* overexpression could be caused by a self-inhibiting mechanism of the pathway, since they are a tumor suppressor and a repressor effector, respectively [26,35]. On the other hand, *SMO* would increase as a result of its activating function at the beginning of the pathway cascade. Considering that *PTCH1* is a known target gene of the pathway [5], our data suggest that *SMO* and *GLI3* increase their expression either directly or indirectly in the assessed signaling cascade. The role of *CCND1* is less evident, as it is elevated in normal tissue and seems to decrease in tumors. Considering its key role in the G1-S phase transition, by increasing cell proliferation, it is likely that other effectors of HH pathway could be also modulated, thus indicating that it is not the only final effector involved in the tumor phenotype.

Perhaps the most interesting and revealing information from our study arises from the analysis of gene expression levels according to the presence of mutations in the *PTCH1* gene. These results show that normal skin with germline mutation releases pathway inhibitors (*PTCH1* and *GLI3*) together with the stimulation of *SMO*. The results in tumors suggest that the suppressors are even more activated with the addition of a mutation in the other allele, generating an effective repression of the *CCND1* effector. This result would indicate that—at least within the gene spectrum assessed in this study—there is a differential regulation in the tumor transformation that depends on the accumulated mutations. Thus, the genetic context would help define the expression profile that leads to such a condition and, consequently, it is extremely useful to know what factors and mechanisms are involved in its modification. It would also be interesting to know if, through the process of tumor transformation, the cell could eventually reach a different phenotype, or if whether the tumoral processes determine treatment outcomes.

In summary, our study explored the physiopathological and genetic understanding of the syndrome. Our findings contribute to knowledge regarding the events taking place inside the cell with an aberrant activation of the HH pathway. This aspect is essential for characterizing the study population, taking into consideration the development and implementation of future prevention and even treatment strategies. Likewise, from these outcomes, we can extrapolate important information to further the understanding of complex cell proliferation and differentiation mechanisms in other cancers and diseases, which apply to embryonic abnormalities, growth injuries, etc., in which the Hedgehog signaling pathway is involved.

## Figures and Tables

**Table 1:** Attributes found in nevoid basal cell carcinoma syndrome probands [8].

Patient	Age	Gender	Major Criteria						Minor Criteria				
			>5 BCCs ^1^ or 1 BCC < 30 years	Odontogenic Keratocysts	≥3 palmoplantar Pits	Falx Cerebri Calcification	Bifurcated Ribs	First-Degree Relative	Macrocephaly	Congenital Abnormalities	Skeletal Abnormalities	Radiological Abnormalities	Ovarian/Cardiac Fibromas
SG1	41	M	X	X	X				X				
SG2	12	M	X	X	X				X		X		
SG3	34	F	X	X				X			X		X
SG6	73	F	X		X			X					
SG7	12	M		X		X		X	X				
SG8	40	M	X	X	X				X				
SG9	68	F	X	X	X			X	X				
SG10	66	F	X	X	X			X	X	X			
SG11	49	F	X	X	X				X				
SG12	59	F	X	X	X			X	X	X			
SG13	55	M	X	X	X				X	X		X	
SG14	70	M	X	X	X			X		X			
SG15	46	M	X	X	X					X			
SG16	51	F	X	X	X			X		X			
SG17	33	F	X	X	X		X	X					
SG18	41	M	X	X	X								
SG19	60	F	X		X					X			
SG20	34	F		X	X			X	X	X			
SG21	14	F	X	X	X			X					
SG22	31	F	X	X	X	X			X				

^1^ BCCs: Basal cell carcinomas.

**Table 2:** Mutations, according to their nature, found in index cases from each family examined.

Patient	Exon	Mutation		Effect on Sequence	Blood	Tumor	Nature
SG1	10 22	c.1392_1405del c.3587C>T	p.(Lys464Asnfs*28) p.(Pro1196Leu)	Frameshift Missense	X –	X X	Germline Somatic
SG2	15	c.2309_2312del	p.(Val771Glufs*34)	Frameshift	X	X	Germline
SG6	19	c.3277G>A [23,24] c.3277G>A	p.(Gly1093Arg) LOH^1^	Missense	X –	X X	Germline Somatic
SG7	10	c.1375_1399dup [12]	p.(Gly467Alafs*38)	Frameshift	X	–	Germline
SG11	3	c.513del	p.(Thr172Glnfs*48)	Frameshift	X	X	Germline
SG12	14 –	c.2012dup [25,26] Allelic deletion	p.(His671Glnfs*10)	Frameshift Allelic loss	X –	X X	Germline Somatic
SG13	Intron7	c.1068-2A>G c.1068-2A>G	– LOH^1^	Frameshift	X –	X X	Germline Somatic
SG17	4 22	c.652dup c.3580C>T	p.(Gln218Argfs*2) p.(Pro1194Ser)	Frameshift Missense	X –	X X	Germline Somatic
SG18	9	c.1286del	p.(Asp429Alafs*3)	Frameshift	X	X	Germline
SG20	3	c.573C>G [13]	p.(Tyr191*)	Nonsense	X	–	Germline
SG21	16	c.2677del	p.(Arg893Alafs*10)	Frameshift	X	X	Germline
SG22	2	c.290del	p.(Asn97Thrfs*20)	Frameshift	X	X	Germline

^1^ LOH: Loss of heterozygosity.
